# Supplementary material for: The self-reference memory bias is preceded by an other-reference bias in infancy
Source: Nat Commun. 2025 Jul 9;16:6311. doi: 10.1038/s41467-025-61642-z (PMC12238355; doi:10.1038/s41467-025-61642-z)
Supplement: Supplementary file 1 — Supplementary Information [file 41467_2025_61642_MOESM1_ESM.pdf]

Supplementary Information (SI)

The self-reference memory bias is preceded by an other-reference bias in infancy

Supplementary Methods (SM)

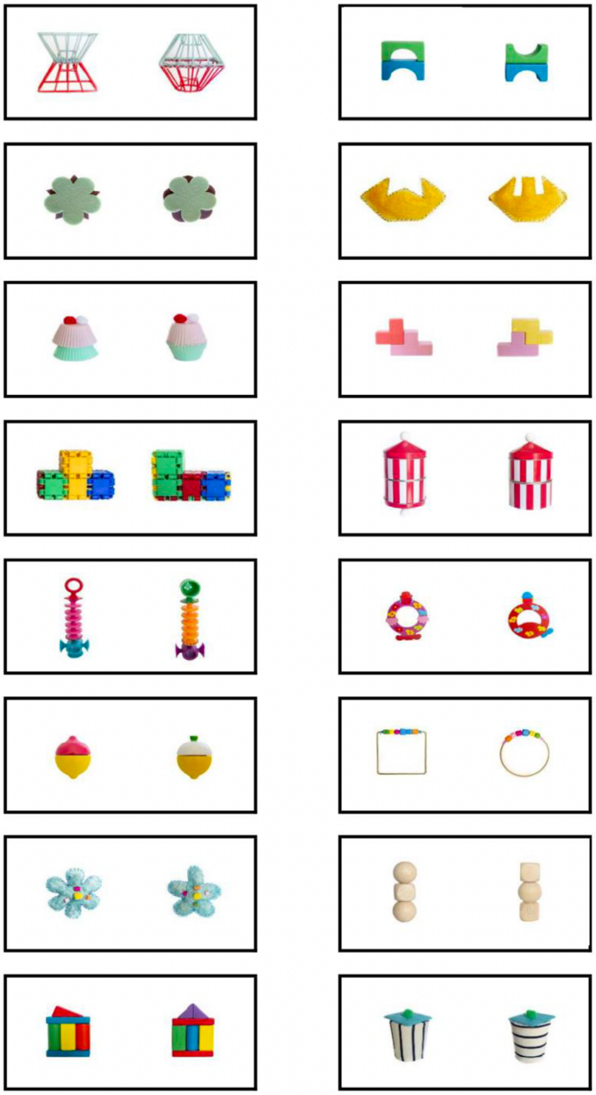

Figure S1. Materials. Set of objects presented during the object encoding phase and their modified counterparts presented alongside these objects in the memory test phase. Which objects were the familiar and which the modified versions was counterbalanced across participants. One column was used as self-assigned objects and the second column as other-assigned objects (counterbalanced across participants).

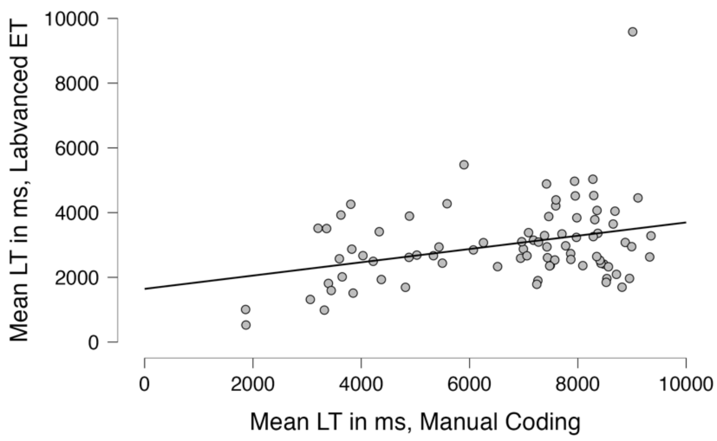

Figure S2. Webcam-based eye-tracking (Experiment 2). Correlation between children's looking times (LT) according to the webcam-based eye-tracking with Labvanced and the manual coding. The hosting platform Labvanced provided webcam-based eye-tracking data consisting of non-equidistant time stamps and (x,y) coordinates. If at least two consecutive coordinates where within the same side of the screen, the difference between the corresponding time stamps was counted as looking time within this side of the screen and accumulated. There was only a weak correlation between the average total looking times based on the webcam data and the manual coding (r = 0.336, BF = 14.83, N = 82). We therefore decided to move forward with the manual coding data (which had high interrater reliability).

SM 1. Analyses with the sign test

Since our data was not normally distributed (see Figure S6 and S7), Wilcoxon signed rank tests were performed. The Wilcoxon signed-rank assumes that the distribution of the differences between the two samples is symmetric, which was not always the case in our data. Therefore, we repeated our main analysis using a sign test that does not assume such symmetry. This yielded highly comparable results. Since the Wilcoxon signed-rank does not only take the signs but also the magnitudes of the differences into account, it is more robust and powerful and is therefore reported in the main manuscript.

Experiment 1. As for the Wilcoxon signed rank test, the sign test yielded moderate evidence against a higher DLS for self-assigned than other-assigned objects in the entire sample (n=73, n_n=33, n_p=40, BF_{10} = 0.320). As before, for mirror recognizers, the sign test provided moderate evidence for a higher DLS for the self-owned than the other-owned objects (n=34, n_n=10, n_p=24, BF_{10} = 7.424). For mirror non-recognizers, the sign test provided strong evidence against a higher DLS for the self-owned

than the other-owned objects (n=37, n_n=23, n_p=14, BF_{10} = 0.085).

In mirror recognizers, the sign test yielded moderate evidence against a positive DLS both for self-owned objects (n=34, n_n=16, n_p=18, BF_{10}=0.282) and the other-owned objects (n=34, n_n=19, n_p=15, BF_{10}=0. 132). In mirror non-recognizers, the sign test provided moderate evidence against a positive DLS for self-owned objects (n=37, n_n=19, n_p=18, BF_{10}=0.178) and moderate evidence for a positive DLS for other-owned objects (n=37, n_n=11, n_p=26, BF_{10}=8.4020).

Experiment 2. As for the Wilcoxon signed rank test, the sign test yielded moderate evidence for a positive DLS for self-owned objects in the online sample (n=82, n_n=31, n_p=51, BF_{10}=3.083), against a positive DLS for other-owned objects (n=82, n_n=42, n_p=40, BF_{10}=0.116) and against a higher DLS for self- than other-owned objects (n=82, n_n=37, n_p=45, BF_{10}=0. 327).

SM S2. Effects of age

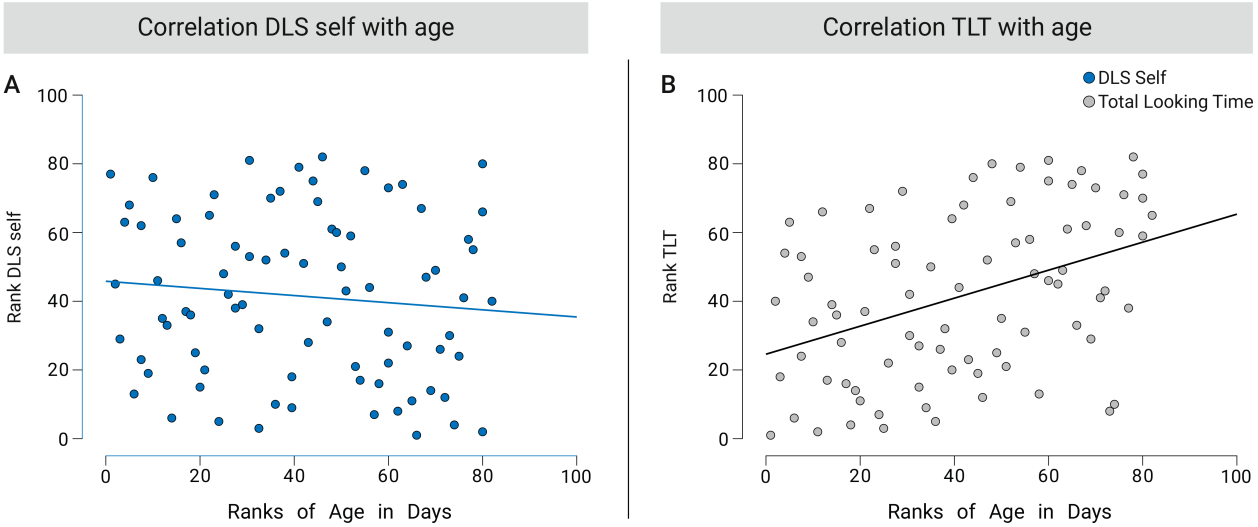

Figure S3. (A) No correlation between the DLS for self-assigned objects and age (Kendall's tau B = –0.073; BF_{10} = 0.077, N = 82), **(B)** Children's total looking times (LTL) to the screen increased with age (Kendall's tau B = 0.273; BF_{10} = 193.8, N = 82).

SM 2.1. Linear mixed model with age as continuous predictor (experiment 2)

We had also preregistered a Bayesian linear mixed model with looking time as the dependent variable, and the factors ownership and stimulus novelty, subject, and age as a continuous predictor. This analysis provided extreme evidence for a main effect of age (BF_{10} = 493.373, error = 1.968%), which reflected the fact that older children looked longer to the screen (for details, see section S6). There was moderate evidence against a main effect of ownership (BF_{10} = 0.243, error = 1.691%, $\eta_p^2 < 0.001$) and inconclusive evidence for a main effect of novelty (BF_{10} = 2.011, error = 3.411%, η_p^2 = 0.017) and the interaction (BF_{10} = 0.813, error = 2.413%, η_p^2 = 0.003).

SM 2.2. Analyses by two age groups (experiment 2)

In experiment 2, we tested toddlers aged 20-40 months to confirm the familiarity effect observed in mirror self-recognizers in experiment 1, and to test whether the observed self-reference effect continues increasing with age after an initial self-concept has developed or remains stable thereafter. No correlation of toddlers' DLS with age was found (see Results section). In addition, we split the sample in two separate equal-sized age groups, yielding similar results as for the entire age range. Specifically, Bayesian ANOVAs on infants' average looking times with the factors *ownership* and *stimulus novelty* provided moderate evidence against a main effect of ownership (young: BF_{10} = 0.285, error = 1.989%, η_p^2 = 0.023; old: BF_{10} = 0.238, error = 1.401%, η_p^2 = 0.013) and remained inconclusive for the main effect of novelty (young: BF_{10} = 1.408, error = 2.05%, η_p^2 = 0.104; old: BF_{10} = 0.435, error = 4.06%, η_p^2 = 0.047) and the interaction (young: BF_{10} = 0.573, error = 8.806%, η_p^2 = 0.034; old: BF_{10} = 0.467, error = 4.578%, η_p^2 = 0.029).

SM 3. Sequential Testing

Experiment 1. As preregistered, we collected a minimum of 70 valid data sets in order to control for false positives and negatives and continued testing children until the BF of the interaction between mirror self-recognition and ownership and BF of the direct comparison between the DLS for self- and the DLS for other-objects in the entire sample reached 3 or 1/3. This was already the case for our minimal N. Since our inclusion criteria involved data preprocessing and manual coding, by the time our analyses converged, we had collected data of three additional children and report the results of the full sample (N = 73).

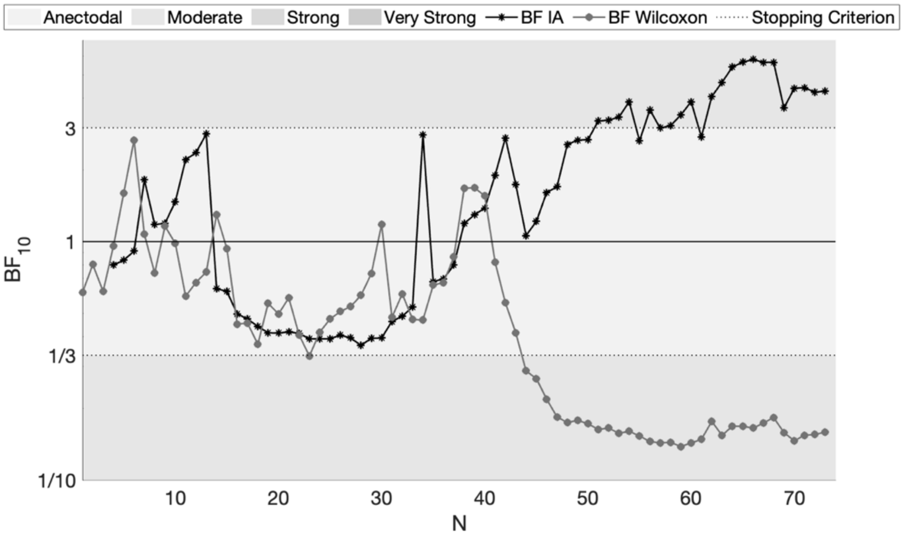

Figure S4. Sequential Bayes factors (BF) for the interaction of mirror self-recognition and ownership in the 2×2 mixed Bayesian ANOVA (BF IA) and the direct comparison between the DLS for self- and other-assigned objects in the entire sample (Wilcoxon signed rank test) by subject ID in experiment 1.

Experiment 2. As preregistered, we collected a minimum of 40 valid data sets to control for false positives and negatives. As we decided to conduct our analyses with the DLS instead of with children's raw looking times for consistency with experiment 1, we adapted our preregistered stopping criterion accordingly and continued testing until evidence for or against a DLS larger 0 converged for both the self-assigned and other-assigned objects with a BF of 3 or 1/3. The DLS for the other-assigned objects converged against an effect at the minimum N of 40, and the DLS for the self-assigned objects converged for an effect at N = 80. An additional 2 children were tested, and we report the data of the full sample of N = 82.

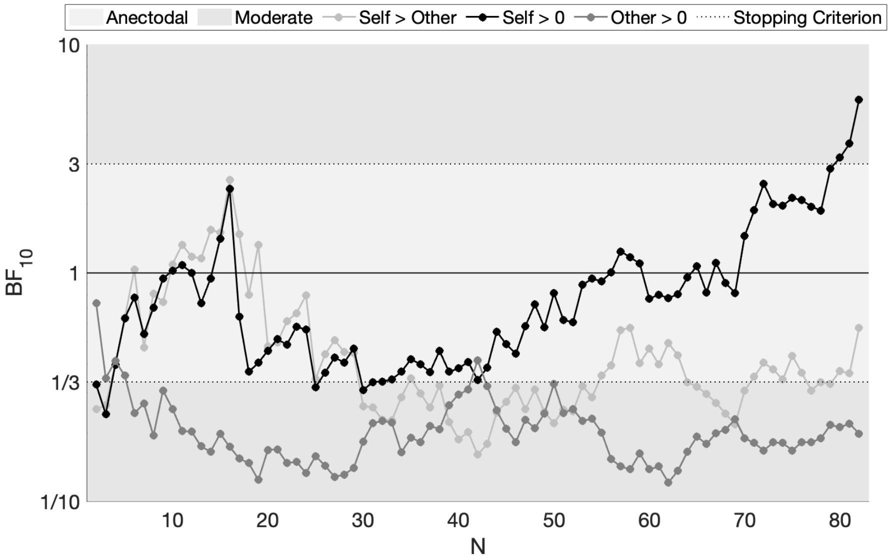

95

Figure S5. Sequential Bayes factors (BF) for Wilcoxon signed rank tests of the DLS for self-assigned objects and other-assigned objects against 0.

SM 4. Distribution of the dependent variable (DLS)

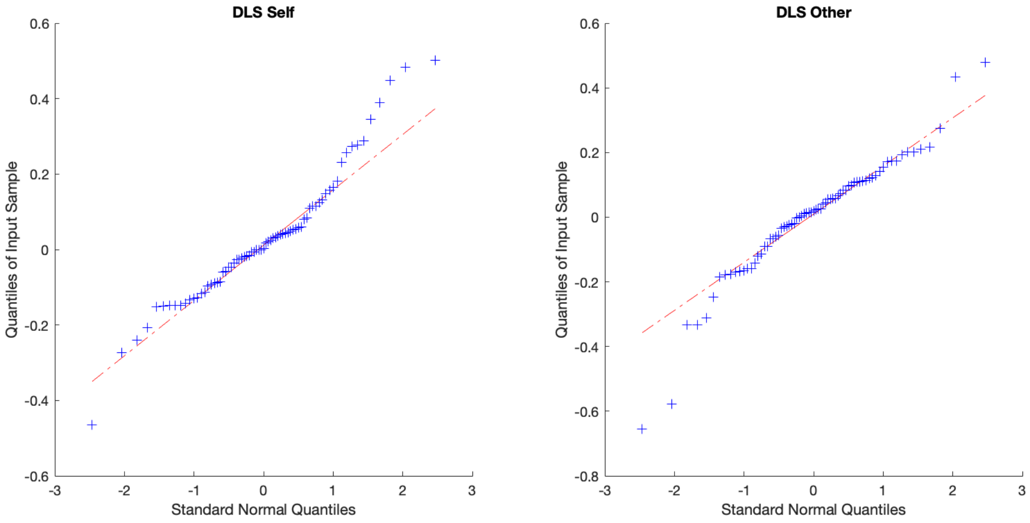

Figure S6. Distribution of the DLS in Experiment 1. As the DLS was not normally distributed, we used non-parametric tests in both experiments whenever feasible.

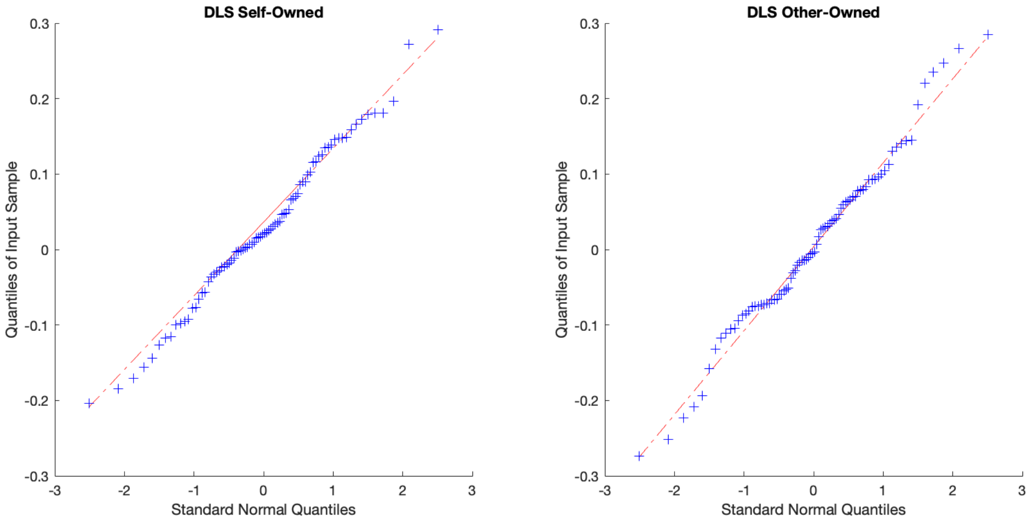

Figure S7. Distribution of the DLS in Experiment 2. As the DLS was not normally distributed, we used non-parametric tests in both experiments whenever feasible.

SM 5. Sex-Related Effects

Experiment 1. In the full sample (N = 73), there was moderate evidence against a sex difference in the DLS for other-owned objects (Bayesian Mann-Whitney U test: BF_{10} = 0.295, W = 570, $\hat{R}$ = 1.003, r_{rb} = 0.136; Male: N = 35, Mean = –0.009, SD = 0.188; Female: N = 37, Mean = 0.011, SD = 0.187) and inconclusive evidence for the DLS for self-owned objects (BF_{10} = 0.465, W = 783, $\hat{R}$ = 1.003, r_{rb} = 0.136; Male: N = 35, Mean = 0.05, SD = 0.01; Female: N = 37, Mean = 0.01, SD = 0.159).

We further tested the sex difference within the recognizers and non-recognizers groups separately. In the recognizers (N = 34), the evidence remained inconclusive for both the DLS for self-owned objects (BF_{10} = 1.912, W = 199, $\hat{R}$ = 1.001, r_{rb} = 0.458; Male: N = 13, Mean = 0.12, SD = 0.174; Female: N = 21, Mean = –0.003, SD = 0.139) and other-owned objects (BF_{10} = 0.365, W = 141, $\hat{R}$ = 1.002, r_{rb} = 0.033; Male: N = 13, Mean = –0.045, SD = 0.225; Female: N = 21, Mean = –0.052, SD = 0.184). In the non-recognizers (N = 36), we found evidence against a sex difference in the DLS for self-owned objects (BF_{10} = 0.331, W = 169, $\hat{R}$ = 1.001, r_{rb} = 0.056; Male: N = 20, Mean = 0.007, SD = 0.192; Female: N = 16, Mean = 0.027, SD = 0.186) and inconclusive evidence for the DLS for the other-owned objects (BF_{10} = 0.77, W = 103, $\hat{R}$ = 1.001, r_{rb} = –0.356; Male: N = 20, Mean = 0.029, SD = 0.152; Female: N = 16, Mean = 0.094, SD = 0.162).

Additionally, there was no evidence for sex difference in the mirror self-recognition test (BF_{10} = 0.812, Cramer's V = 0.173, Bayesian Contingency Table; Male: 13/33 passers, Female: 21/37 passers).

Experiment 2. Similarly, there was moderate evidence against sex differences in the DLS for other-owned objects (BF_{10} = 0.250, W = 789, $\hat{R}$ = 1.015, r_{rb} = –0.024; Male: N = 33, Mean = –0.002, SD = 0.131; Female: N = 49, Mean = 0.012, SD = 0.101) and inconclusive evidence for self-owned objects (BF_{10} = 0.487, W = 661, $\hat{R}$ = 1.003, r_{rb} = –0.088; Male: N = 33, Mean = 0.009, SD = 0.119; Female: N = 49, Mean = 0.042, SD = 0.089).

That is, overall, no evidence for differences by sex were found in either of the two experiments. Note, however, that the study was not designed to test for sex effects, so that the subgroups of male/female mirror recognizers versus non-recognizers were too small to yield conclusive evidence.

Supplementary Note 1

No differences in inhibition between mirror recognizers and non-recognizers

As N = 44 of the infants in experiment 1 also participated in a different study, we had access to their performance on the Early Childhood Inhibition Touch Screen Task (ECITT, Holmboe et al. 2021), which we took to be a proxy of their cognitive maturity. The task was presented on an 11” Apple iPad tablet, which the experimenter held in front of the child who was seated on their parent’s lap. Children saw two buttons on the screen and were instructed to “press the happy face”. The task began with 4 practice trials in which a single blue “smiley” icon was presented in the center of the screen. On the first practice trial, the experimenter demonstrated that tapping this icon would result in a short cartoon animation with sound effects. On the second trial the child was encouraged to “press the happy face”. If the child was reluctant to press the button, the experimenter demonstrated it again (for up to 3 more trials) until the child was happy to press the button on their own.

After the fourth practice trials, a single block of 32 experimental trials were presented. On each trial two buttons were shown on the screen and the child was instructed to “press the happy face”. If the child tapped the correct button, an animation played, after which the next trial began immediately. If the child tapped the incorrect button, the buttons disappeared from the screen for 1s before the next trial began. The smiley button appeared in the prepotent location on 75% of trials (24 trials) and in the inhibitory location on 25% of trials (8 trials). The experimental blocks always began with at least 3 prepotent trials. On the first trial the experimenter pointed to the correct response location to ensure that the child responded to the prepotent location from the beginning; this first trial was always removed from the analysis. To ensure a high level of participant engagement, the experimenter reminded the child to “press the happy face” as needed throughout the testing session and provided encouraging comments when the reward animations were shown.

Performance was measured based on accuracy, with a higher score indicating lower inhibitory control. Accuracy was measured by computing an accuracy difference score (ADS) based on subtracting the percentage correct on inhibitory trials from the percentage correct on the prepotent trials. Trials with reaction times shorter than 300ms were excluded following Holmboe et al. (2021). Participants were also excluded if they did not understand or cooperate with the task instructions, based on the criterion of more than 60% correct on prepotent trials.

After these exclusions, we retained inhibitory control data from 29 infants (n = 16 non-recognisers and n = 13 recognisers). As expected, infants were more accurate on prepotent

trials compared to inhibition trials (proportion correct prepotent trials = 0.79 ± 0.20, proportion correct inhibition trials = 0.51 ± 0.19; Bayesian Wilcoxon signed rank test BF_{10} = 1122.44, W = 5, R = 1.213), and there was no evidence for a difference in performance between mirror recognisers and non-recognisers based on accuracy on prepotent trials (proportion correct prepotent trials in non-recognizers = 0.78 ± 0.18; in recognizers = 0.79 ± 0.22, Bayesian Mann-Whitney test, BF_{10} = 0.36, W = 100.5, R = 1.001), accuracy on inhibition trials (proportion correct inhibition trials in non-recognizers = 0.53 ± 0.17, in recognizers = 0.48 ± 0.23, Bayesian Mann-Whitney test, BF_{10} = 0.42, W = 124.5, R = 1.001), or accuracy difference score between prepotent and inhibition trials score (non-recognizers = 0.21 ± 0.09, recognizers = 0.24 ± 0.25, Bayesian Mann-Whitney test, BF_{10} = 0.43, W = 86.5, R = 1.003).

There was also evidence against a difference in inhibitory control between mirror recognizers and non-recognizers in the full sample of N = 48 children who were included in the ECITT and mirror test (n = 27 non-recognizers and n = 21 recognizers; proportion correct prepotent trials in non-recognizers = 0.76 ± 0.19 and in recognizers = 0.81 ± 0.18, Bayesian Mann-Whitney test, BF_{10} = 0.35, W = 242.5, R = 1.001; proportion correct inhibition trials in non-recognizers = 0.50 ± 0.19 and in recognizers = 0.49 ± 0.21, Bayesian Mann-Whitney test, BF_{10} = 0.30, W = 301, R = 1.002; accuracy difference score in non-recognizers = 0.22 ± 0.13 and in recognizers = 0.26 ± 0.22, Bayesian Mann-Whitney test, BF_{10} = 0.34, W = 239, R = 1.004).
