# Supplementary material for: The self-reference memory bias is preceded by an other-reference bias in infancy
Source: Nat Commun. 2025 Jul 9;16:6311. doi: 10.1038/s41467-025-61642-z (PMC12238355; doi:10.1038/s41467-025-61642-z)
Supplement: Supplementary file 2 — Reporting Summary [file 41467_2025_61642_MOESM2_ESM.pdf]

nature portfolio

Corresponding author(s): Charlotte Grosse Wiesmann
Max Planck Institute for Human Cognitive and Brain Sciences, Leipzig
wiesmann@cbs.mpg.de

Last updated by author(s): 2025.04.19

Reporting Summary

Nature Portfolio wishes to improve the reproducibility of the work that we publish. This form provides structure for consistency and transparency in reporting. For further information on Nature Portfolio policies, see our Editorial Policies and the Editorial Policy Checklist.

Statistics

For all statistical analyses, confirm that the following items are present in the figure legend, table legend, main text, or Methods section.

n/a	Confirmed	
☐	☒	The exact sample size (*n*) for each experimental group/condition, given as a discrete number and unit of measurement
☐	☒	A statement on whether measurements were taken from distinct samples or whether the same sample was measured repeatedly
☐	☒	The statistical test(s) used AND whether they are one- or two-sided<br>*Only common tests should be described solely by name; describe more complex techniques in the Methods section.*
☐	☒	A description of all covariates tested
☐	☒	A description of any assumptions or corrections, such as tests of normality and adjustment for multiple comparisons
☐	☒	A full description of the statistical parameters including central tendency (e.g. means) or other basic estimates (e.g. regression coefficient) AND variation (e.g. standard deviation) or associated estimates of uncertainty (e.g. confidence intervals)
☐	☒	For null hypothesis testing, the test statistic (e.g. *F*, *t*, *r*) with confidence intervals, effect sizes, degrees of freedom and *P* value noted<br>*Give P values as exact values whenever suitable.*
☐	☒	For Bayesian analysis, information on the choice of priors and Markov chain Monte Carlo settings
☒	☐	For hierarchical and complex designs, identification of the appropriate level for tests and full reporting of outcomes
☐	☒	Estimates of effect sizes (e.g. Cohen's *d*, Pearson's *r*), indicating how they were calculated

Our web collection on statistics for biologists contains articles on many of the points above.

Software and code

Policy information about availability of computer code

Data collection	We used the software Experiment Builder and Labvanced to present the stimuli and record eye-tracking data.
Data analysis	We used the software Boris version 7.13 to manually code video data, and Matlab 2023b, JASP 0.18.3, R Studio 2024.04.0 for statistical data analysis and visualization.

For manuscripts utilizing custom algorithms or software that are central to the research but not yet described in published literature, software must be made available to editors and reviewers. We strongly encourage code deposition in a community repository (e.g. GitHub). See the Nature Portfolio guidelines for submitting code & software for further information.

Data

Policy information about availability of data

All manuscripts must include a data availability statement. This statement should provide the following information, where applicable:

- Accession codes, unique identifiers, or web links for publicly available datasets
- A description of any restrictions on data availability
- For clinical datasets or third party data, please ensure that the statement adheres to our policy

The anonymized coded behavioral data generated in this study have been deposited at OSF under https://doi.org/10.17605/OSF.io/Z3T8F.[40] The raw video data of the infants are protected and are not available due to data protection regulations. Further, several participants in Experiment 2 did not consent to data sharing, so that only a subset of this data is available.

Research involving human participants, their data, or biological material

Policy information about studies with human participants or human data. See also policy information about sex, gender (identity/presentation), and sexual orientation and race, ethnicity and racism.

Reporting on sex and gender	We aimed at collecting an approximately equal sex distribution in both experiments. Experiment 1: 38 female, 35 male. Experiment 2: 49 female, 33 male. Sex was recorded according to parental report.<br>No evidence for effects of sex on the results were found as reported in the SI section S11.
Reporting on race, ethnicity, or other socially relevant groupings	Participants in experiment 1 were Danish-speaking infants from the Copenhagen area, and in experiment 2 German-speaking children predominantly from the area of Leipzig. The participants were representative of the population in these areas.
Population characteristics	Participants were typically developing infants, aged 17-21 months in experiment 1 and aged 20-40 months in experiment 2.
Recruitment	Children were recruited through the data base of the respective child lab and through the online platform Kinderschaffenwissen.de. Recruitment for the data base is primarily done by sending out letters to all families of newborn infants in the area of Copenhagen and Leipzig respectively. This approach covers the broad population of these cities.
Ethics oversight	Experiment 1 was approved by the Ethics Committee of the Faculty of Social Sciences at Copenhagen University. Experiment 2 was approved by the Ethics Committee of the Medical Faculty at the University of Leipzig.

Note that full information on the approval of the study protocol must also be provided in the manuscript.

Field-specific reporting

Please select the one below that is the best fit for your research. If you are not sure, read the appropriate sections before making your selection.

☐ Life sciences ☒ Behavioural & social sciences ☐ Ecological, evolutionary & environmental sciences

For a reference copy of the document with all sections, see nature.com/documents/nr-reporting-summary-flat.pdf

Behavioural & social sciences study design

All studies must disclose on these points even when the disclosure is negative.

Study description	The study is quantitative experimental and cross-sectional.
Research sample	see above
Sampling strategy	Sample sizes were determined according to a preregistered Bayesian sequential testing scheme with a minimum sample of N=70 (Experiment 1) and N=40 (Experiment 2) until moderate evidence for or against our main hypothesis was found (corresponding to a Bayes Factor >3 or <1/3).
Data collection	Data was recorded with an Eyelink1000 Plus eye-tracker in experiment 1 and with a webcam through the platform Labvanced in experiment 2.<br>Infants were seated on their parent's lap throughout the experiment.<br>There were no experimental groups.
Timing	Data collection lasted from October 2020 - August 2023.
Data exclusions	In experiment 1, 6 infants were excluded because they did not provide at least 2 self-assigned and 2 other-assigned object trials.<br>In experiment 2, N=36 children had to be excluded because of severe technical problems with the hosting platform Labvanced leading to no or insufficient recorded data (criteria as in experiment 1), N=8 because of missing parental report on mirror self-recognition, and N=1 because of not recognizing themselves in the mirror.
Non-participation	6 infants dropped out because they were distracted or aborted the experiment, leading to less than 2 self-owned object and 2 other-owned object trials (see above).
Randomization	Participants were not allocated into experimental groups, but all saw the same stimuli in different trial orders randomized across participants.

Reporting for specific materials, systems and methods

We require information from authors about some types of materials, experimental systems and methods used in many studies. Here, indicate whether each material, system or method listed is relevant to your study. If you are not sure if a list item applies to your research, read the appropriate section before selecting a response.

Materials & experimental systems

n/a	Involved in the study
☒	☐ Antibodies
☒	☐ Eukaryotic cell lines
☒	☐ Palaeontology and archaeology
☒	☐ Animals and other organisms
☒	☐ Clinical data
☒	☐ Dual use research of concern
☒	☐ Plants

Methods

n/a	Involved in the study
☒	☐ ChIP-seq
☒	☐ Flow cytometry
☒	☐ MRI-based neuroimaging

Plants

Seed stocks

n.a.

Novel plant genotypes

n.a.

Authentication

n.a.
